# Acceptability of a Web-Based Health App (PortfolioDiet.app) to Translate a Nutrition Therapy for Cardiovascular Disease in High-Risk Adults: Mixed Methods Randomized Ancillary Pilot Study

**DOI:** 10.2196/58124

**Published:** 2025-03-28

**Authors:** Meaghan E Kavanagh, Laura Chiavaroli, Selina M Quibrantar, Gabrielle Viscardi, Kimberly Ramboanga, Natalie Amlin, Melanie Paquette, Sandhya Sahye-Pudaruth, Darshna Patel, Shannan M Grant, Andrea J Glenn, Sabrina Ayoub-Charette, Andreea Zurbau, Robert G Josse, Vasanti S Malik, Cyril W C Kendall, David J A Jenkins, John L Sievenpiper

**Affiliations:** 1 Department of Nutritional Sciences Temerty Faculty of Medicine University of Toronto Toronto, ON Canada; 2 Toronto 3D Knowledge Synthesis and Clinical Trials Unit, Clinical Nutrition and Risk Factor Modification Center St. Michael’s Hospital Unity Health Toronto Toronto, ON Canada; 3 Li Ka Shing Knowledge Institute St. Michael’s Hospital Unity Health Toronto Toronto, ON Canada; 4 Departments of Pediatrics and Obstetrics and Gynaecology IWK Health Halifax, NS Canada; 5 Department of Obstetrics and Gynaecology Faulty of Medicine Dalhousie University Halifax, NS Canada; 6 Department of Nutrition and Food Studies New York University New York, NY United States; 7 Department of Nutrition Harvard T.H. Chan School of Public Health Boston, MA United States; 8 Division of Endocrinology and Metabolism St. Michael’s Hospital Unity Health Toronto Toronto, ON Canada; 9 Department of Medicine Temerty Faculty of Medicine University of Toronto Toronto, ON Canada

**Keywords:** diet, apps, dietary app, Portfolio Diet, dietary portfolio, cholesterol reduction, cardiovascular disease, eHealth, usability, acceptability

## Abstract

**Background:**

The Portfolio Diet is a dietary pattern for cardiovascular disease (CVD) risk reduction with 5 key categories including nuts and seeds; plant protein from specific food sources; viscous fiber sources; plant sterols; and plant-derived monounsaturated fatty acid sources. To enhance implementation of the Portfolio Diet, we developed the PortfolioDiet.app, an automated, web-based, multicomponent, patient-facing health app that was developed with psychological theory.

**Objective:**

We aimed to evaluate the effect of the PortfolioDiet.app on dietary adherence and its acceptability among adults with a high risk of CVD over 12 weeks.

**Methods:**

Potential participants with evidence of atherosclerosis and a minimum of one additional CVD risk factor in an ongoing trial were invited to participate in a remote web-based ancillary study by email. Eligible participants were randomized in a 1:1 ratio using a concealed computer-generated allocation sequence to the PortfolioDiet.app group or a control group for 12 weeks. Adherence to the Portfolio Diet was assessed by weighed 7-day diet records at baseline and 12 weeks using the clinical Portfolio Diet Score, ranging from 0 to 25. Acceptability of the app was evaluated using a multifaceted approach, including usability through the System Usability Scale ranging from 0 to 100, with a score >70 being considered acceptable, and a qualitative analysis of open-ended questions using NVivo 12.

**Results:**

In total, 41 participants were invited from the main trial to join the ancillary study by email, of which 15 agreed, and 14 were randomized (8 in the intervention group and 6 in the control group) and completed the ancillary study. At baseline, adherence to the Portfolio Diet was high in both groups with a mean clinical Portfolio Diet Score of 13.2 (SD 3.7; 13.2/25, 53%) and 13.7 (SD 5.8; 13.7/25, 55%) in the app and control groups, respectively. After the 12 weeks, there was a tendency for a mean increase in adherence to the Portfolio Diet by 1.25 (SD 2.8; 1.25/25, 5%) and 0.19 (SD 4.4; 0.19/25, 0.8%) points in the app and control group, respectively, with no difference between groups (*P*=.62). Participants used the app on average for 18 (SD 14) days per month and rated the app as usable (System Usability Scale of mean 80.9, SD 17.3). Qualitative analyses identified 4 main themes (user engagement, usability, external factors, and added components), which complemented the quantitative data obtained.

**Conclusions:**

Although adherence was higher for the PortfolioDiet.app group, no difference in adherence was found between the groups in this small ancillary study. However, this study demonstrates that the PortfolioDiet.app is considered usable by high-risk adults and may reinforce dietitian advice to follow the Portfolio Diet when it is a part of a trial for CVD management.

**Trial Registration:**

ClinicalTrials.gov NCT02481466; https://clinicaltrials.gov/study/NCT02481466

## Introduction

### Background

Cardiovascular disease (CVD) remains the leading cause of death globally [1]. Effective prevention and management strategies are needed to target modifiable risk factors for CVD. Several recent Canadian population-based studies have shown that many patients at high CVD risk continue to have low-density lipoprotein cholesterol (LDL-C) levels well above the guideline targets [2,3]. LDL-C has been extensively studied and described as a causal factor for CVD [4]. LDL-C levels above the target can result from multiple factors such as insufficient LDL-C lowering with statins, statin-related side effects, suboptimal medication adherence, and treatment inertia [5]. Amid these challenges, dietary approaches for CVD risk reduction emerge as a potentially powerful tool [6] with clinical practice guidelines universally recommending diet and lifestyle as the cornerstone of therapy for addressing CVD [7,8].

The Portfolio Diet is a dietary pattern recognized by clinical practice guidelines in Canada and internationally, including the Canadian Cardiovascular Society [7,8] Diabetes Canada [9], Obesity Canada [10], Canadian Cardiovascular Harmonized National Guidelines Endeavour [11], Heart UK [12], European Atherosclerosis Society [13], and the American College of Cardiology and American Heart Association guidelines [14]. The Portfolio Diet has been shown to have the same LDL-C and inflammatory (C-reactive protein) reductions (approximately 30%) as statin therapy in a head-to-head randomized controlled trial in participants with hyperlipidemia [15]. A systematic review and meta-analysis of clinical trials [16] confirmed these “drug-like” effects and demonstrated clinically meaningful cardiovascular benefits on other targets including non–high-density lipoprotein cholesterol, apolipoprotein B, triglycerides, blood pressure, and estimated 10-year CVD risk.

Although the Portfolio Diet, among other dietary patterns, is recognized in guidelines, uptake and implementation of nutrition therapies in clinical practice remains limited. This dilemma stems from several barriers that hinder the widespread adoption of nutrition therapies. Chief among these challenges are the shortage of available health support services, the restricted access to registered dietitians, the time constraints faced by physicians, and the lack of comprehensive education and tools [17,18]. The resulting consequence of these obstacles is that many patients who would benefit from nutrition therapy do not receive it [19]. In a survey of Canadian patients randomly selected from family health networks, only 37% reported receiving nutrition counseling in primary care [20], highlighting the need for effective dissemination strategies.

Due to their highly scalable nature, the use of technology to aid in the dissemination and delivery of lifestyle behavior change interventions has become of great interest with the number of studies investigating health apps having gone up rapidly since 2010 [21]. Web- and mobile-based applications (hereafter apps) provide an important alternative and complementary approach for the delivery and long-term reinforcement of health advice. Previous work has found that apps can be a cost-effective method for the delivery of lifestyle interventions such as in smoking cessation [22,23]. As smartphones become common everyday household items, the possible reach and impact of using apps to deliver interventions grows. Currently, it is estimated that over 300,000 health apps exist on app stores [24]; however, most publicly available health apps remain untested.

### Objective

To enhance the implementation of the Portfolio Diet in health care settings, we developed the PortfolioDiet.app [25], a free, web-based, multicomponent, patient-facing engagement and educational tool. While we have previously undertaken quality improvement and usability testing of the PortfolioDiet.app in a convenience sample [26], the app has not yet been evaluated in its intended population of adults at high risk of CVD. Therefore, the objective of this study was to evaluate the effect of the PortfolioDiet.app on dietary adherence and its acceptability among adults with a high risk of CVD over 12 weeks.

## Methods

### Design

This mixed methods ancillary study was a 12-week single-center, open label, randomized controlled ancillary study within an ongoing 3-year multicenter randomized controlled trial, the Combined Portfolio Diet and Exercise Study (PortfolioEx; ClinicalTrials.gov NCT02481466). All participants for the ancillary study were recruited from those randomized to one of the 2 Portfolio Diet arms at the St. Michael’s Hospital, Toronto, Canada, site of the main trial. Recruited participants were randomized to receive the PortfolioDiet.app for 12 weeks or to the control group.

We used a mixed methods approach where we collected and analyzed both quantitative and qualitative data to ensure a thorough and comprehensive assessment of the intervention [27]. Multimedia Appendix 1 shows the CONSORT (Consolidated Standards of Reporting Trials) checklist and Multimedia Appendix 2 shows the CONSORT‐EHEALTH (Consolidated Standards of Reporting Trials of Electronic and Mobile Health Applications and Online Telehealth) checklist (version 1.6.1) [28]. Our intention was to gather complementary data from quantitative and qualitative methods and then integrate findings within a data triangulation design [29], enabling us to draw metainferences regarding the acceptability and usability of the PortfolioDiet.app. These insights will not only inform potential refinements to the app itself but also guide the design of a future trial.

### Ethical Considerations

The ancillary study was conducted according to the guidelines of the Declaration of Helsinki and approved as an ancillary study to the main PortfolioEx trial by the research ethics board (REB) of St. Michael’s Hospital, Unity Health Toronto (REB 14-316). All participants provided written informed consent to the main trial and separately provided verbal over-the-phone informed consent to the ancillary study. No compensation was provided to the participants. Participant data were stored at St. Michael’s Hospital and kept confidential by ensuring identifying data were kept separate from study data using a study ID. All study data were deidentified, and the master linking log was kept in a password-protected file on a secure drive at St. Michael’s Hospital, only accessible to study staff.

### Study Participants

Participants in the PortfolioEx trial were men and postmenopausal women with a BMI ≤40 kg/m^2^ who were considered at high risk for CVD. Participants had carotid artery plaque buildup (an intima-media thickness of ≥1.2 mm) in addition to at least one other of the following characteristics: type 2 diabetes, history of myocardial infarction or angioplasty, hypercholesterolemia and treated with statins or prescribed statins but due to statin intolerance or choice are not taking statins, or raised blood pressure (>140/90 mm Hg). To be eligible for the ancillary study, participants needed to have access to a web portal through a personal smartphone, tablet, or home computer and needed to have an active email address.

### Randomization

Eligible participants were randomized in a 1:1 ratio to either the PortfolioDiet.app group or a control group using a computer-generated allocation sequence. Randomization was done using block sizes of 4 with stratification by sex (ie, male and female), age (ie, <65 and ≥65 years), and their allocated exercise group (ie, yes and no) in the 3-year PortfolioEx trial. MK was responsible for contacting and enrolling participants, providing them with information on the study, and sending them app details and questionnaires. The randomization table was developed from Sealed Envelope [30]. SA-C, who had no contact with participants, was the only one to have access to the randomization table and was responsible for assigning participants to the interventions.

### Theoretical and Operational Design Components of the PortfolioDiet.app

**Figure 1:**
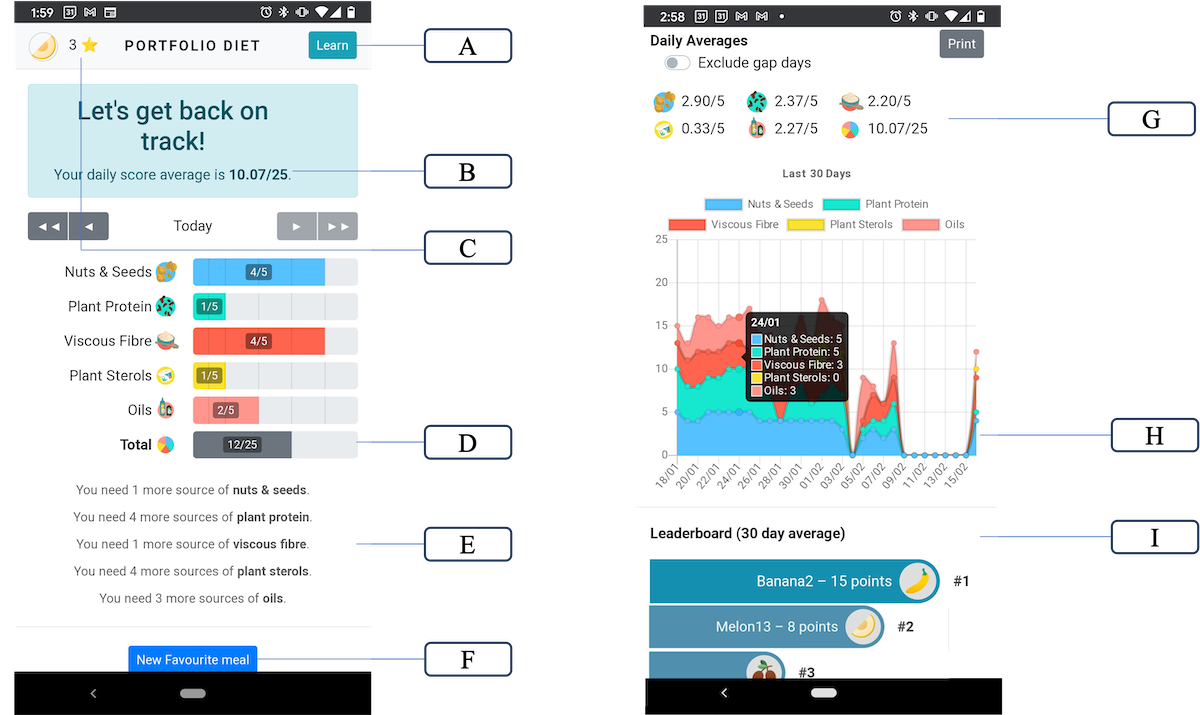
PortfolioDiet.app dashboard with key features highlighted, top to bottom: (A) learn tab that has recipes, tipsheets, and videos; (B) daily average Portfolio Diet Score per month; (C) star rewards, a form of reward for logging into the app and for completing the question of the week; (D) current day total Portfolio Diet Score; (E) specific daily messages related to goals; (F) personal favorite meals for easy tracking; (G) subcategory Portfolio Diet Scores with daily targets of 5 points; (H) progress-tracking graph displaying the monthly progress of the score; and (I) leaderboard with other app users' 30-day average.

The app was developed with integration of the psychological theory including the social cognitive theory and self-regulatory principles of behavior change by providing multiple forms of behavioral feedback on dietary adherence, including tip sheets for promoting dietary change. Designed with a variety of elements to enhance and sustain behavior change, Figure 1 shows the PortfolioDiet.app’s home page with key features highlighted. These include features previously identified as elements preferred by health app users, including a personalized dashboard, goal setting, educational features, and email messages.

Within the “Learn” section, the app houses educational resources including a bank of recipes, tip sheets, and videos (Figure 1). The PortfolioDiet.app offers users an array of recipes that span from family friendly dinner recipes to quick snacks while also including culturally adapted recipes (eg, African, Mediterranean, and South Asian) and filters for dietary restrictions (eg, gluten free, low carbohydrates, and low sodium).

Many of the features would fall under the definition of gamification, which evidence from a systematic review and meta-analysis has found to support behavior change, increasing measures of physical activity and decreasing measures of adiposity [31]. These features include star rewards for engaging with the app, allowing users to track and visualize their average adherence and progress, provides daily goals, and a social competitive aspect through a leaderboard on diet adherence (Figure 1). Star rewards allow users to earn and collect stars, incentivizing users to interact with the app. Users can collect stars by logging into the app and correctly answering the question of the week. The leaderboard feature provides users with an overview of the number of app members and their average daily score over 30 days, allowing users to compare their average daily score with other users.

The app uses a dietary score to guide users on the amount of key foods to eat and to provide personalized feedback. The clinical Portfolio Diet Score (c-PDS) has previously been validated in a similar population of adults with hyperlipidemia [32]. By following the Portfolio Diet, users can earn up to 5 points from each category of Portfolio Diet foods for a maximum c-PDS of 25 points per day in the app. It has previously been shown that an increase in c-PDS by 12 points predicts about a 0.53 mmol/L (13%) reduction in LDL-C in patients with hyperlipidemia over 6 months [32].

When using the PortfolioDiet.app, users can input Portfolio Diet foods and portion sizes. Each food item is shown as 1 portion size, in grams or as cup measurements, with targets based on 1 of 3 caloric levels. The user picks the portion size that is most similar to their intake and then the item will appear on their dashboard. The app allows for self-monitoring and provides feedback through an average daily score on the home page and the current day’s score and, below, a graph displaying the monthly progress for dietary adherence (Figure 1).

### Intervention

Participants randomized to receive the PortfolioDiet.app were sent an instructional guide and videos by email, with instructions on how to create an account and use the app features. The PortfolioDiet.app is fully automated and was provided as a web-based version that could be used on laptops, tablets, smartphones, or public computers such as those in libraries. The dietary advice on the Portfolio Diet conveyed through the app included recommendations on the 5 core cholesterol-lowering foods and their recommended amounts per day for a 2000 kcal diet: 45 g nuts and seeds (eg, tree nuts, peanuts, or seeds); 50 g of plant protein (eg, from soy and dietary pulses); 20 g viscous soluble fiber (eg, from sources such as oats, barley, psyllium, eggplant, okra, apples, oranges, or berries); 2 g plant sterols (eg, from supplements and fortified foods); and 45 g monounsaturated fatty acids (eg, from cold-pressed olive, canola, soybean, “high-oleic” sunflower and safflower oils, or avocados).

Development of the app was frozen during the trial. Participants randomized to the PortfolioDiet.app group were asked to use the app every day (ie, including both weekdays and weekends) over the 12-week intervention in the ancillary study. If a day was missed, participants were encouraged in the app to retroactively enter their Portfolio Diet foods. If participants did not make an account during the first week, they were sent an email reminder every week. Participants were not blinded to their allocation and neither were the study staff. Participants randomized to the control group were informed of their randomization allocation but received no further contact from the PortfolioDiet.app staff until after the study, at which point they were offered access to the app. The 12-week intervention length was chosen to allow for a controlled assessment of the health app on dietary adherence (the main objective), without unfairly restricting access to the app for those participants randomized to not receive the app within an active trial.

As REB approval for this ancillary study was received during the COVID-19 publicly declared emergency (ie, the pandemic). Staff were not permitted to access Unity Health sites or to have in-person contact with participants or staff. Therefore, all study interactions with participants for the study took place over the phone or by email. The interactions in the ancillary study did not provide any dietary counseling support and only provided minimal-contact administrative support to those randomized to the PortfolioDiet.app group, including encouraging the use of materials provided to help with account creation and using the app features.

### Outcomes

The primary outcome was a change in dietary adherence to the Portfolio Diet over 12 weeks in those randomized to the PortfolioDiet.app group compared to those in the control group. Adherence to the Portfolio Diet was assessed from weighed 7-day diet records (7DDRs) collected at baseline and at 12 weeks through predesigned paper-based templates. Participants were trained and supported by registered dietitians to complete the records, and paper copies were mailed to participants with telephone discussions scheduled every 3 months. The c-PDS was calculated from the 7DDRs and ranges from 0 to 25 points, with a score of 0 indicating no adherence to the Portfolio Diet and a score of 25 indicating full adherence to the diet.

Acceptability of the PortfolioDiet.app was assessed in participants who were randomized to the PortfolioDiet.app group. App use was evaluated through the app’s web-based repository based on participants’ log-ins over the 12 weeks. Usability was assessed using the System Usability Scale (SUS). The SUS is a validated usability questionnaire that has been used in clinical settings to assess the usability of various systems and tools [33,34]. The SUS includes 10 statements rated on a 5-point Likert scale (from 1=strongly disagree to 5=strongly agree). The score ranges from 0 to 100 with a score higher than 70 being considered acceptable [35]. We also collected the c-PDS data from the app, which were based on participants’ logged entries into the app. The c-PDS was saved in the app’s web-based repository and, unlike the primary outcome of dietary adherence, was not calculated from the 7DDR.

Multimedia Appendix 3 shows the structured questionnaire used with open-ended questions. The questionnaire collected participant feedback on acceptability, knowledge acquisition, and app features. It was developed by MEK, LC, and SMG using existing tools [36] and included the SUS questionnaire [33]. The questionnaire was emailed to participants after 12 weeks of using the PortfolioDiet.app. Participants were instructed to complete the questionnaire by typing out their responses and to return it by email.

### Analytic Techniques

As part of the primary 3-year PortfolioEx trial, eligibility by intima-media thickness was measured by B-mode Carotid Ultrasound at 12 carotid artery segments (1-cm long) of the near and far walls of the internal, bifurcation, and common left and right carotid arteries. Baseline serum lipids were measured on fasting serum and analyzed in the routine hospital laboratory using Beckman SYNCHRON LX Systems. The LDL-C level was calculated using the Friedewald equation [37]. Anthropometric data were collected when participants were fasting by trained study staff, and information on medications and the diagnosis of type 2 diabetes was collected through self-report questionnaires.

### Analyses

Baseline characteristics were assessed by 2-sample *t* tests for continuous variables and Fisher exact test for categorical variables. Dietary adherence to the Portfolio Diet from weighed 7DDRs measured by the c-PDS (week 0 to week 12) was expressed as mean differences with SDs. Within-group and between-group differences were assessed using a 2-sample *t* test. On the basis of a prior multi-center randomized controlled trial, a total of 56 participants were required to detect a ≥3.28 point difference in c-PDS with 80% power (1-β), ⍺=.05, and SD 4.30 [38]. Statistical analysis was performed using Stata (version 7; StataCorp). The planned sample size of 56 participants, with approximately 23 receiving the app, was deemed sufficient to reach data saturation, particularly given our homogeneous study population, and aligned with the study by Hennink and Kaiser [39], who suggest that smaller sample sizes can be adequate for achieving saturation in qualitative research with homogeneous groups.

For the qualitative analysis, open-ended survey data were extracted from the structured questionnaire and initially analyzed independently using NVivo (version 12.7.0; QSR International) by members of the research team (MEK, LC, SMQ, and GV). The team used reflexive thematic analysis, as described in the study by Braun and Clarke [40], to identify patterns and concepts within the data [40]. A coding framework was collaboratively established, and each member of the research team conducted an individual review of both the data and the coding framework to confirm the accuracy of the interpretations during initial analysis, and to identify any elements or insights that might not have been initially captured during the group analysis. Regular team meetings were held weekly over a 1-month period to discuss coding findings, address discrepancies, and reach consensus on the identified codes. Identified codes were further structured into main themes and subthemes, and a table was produced to arrange quotations derived from the survey responses to substantiate the themes and subthemes identified.

The analysis process was performed with consideration of the trustworthiness criteria [40]. To ensure credibility, dependability, confirmability, and transferability in the qualitative analysis, multiple researchers were involved in the coding process to reach consensus on identified themes, a detailed description of coding decisions and theme development was maintained, and potential biases were acknowledged with regular discussions to minimize influence. In addition, a detailed description of the study, participants, and findings was provided to enable readers to assess the applicability of the results to other settings.

After both analyses were conducted, the qualitative findings were compared with the quantitative results using a data triangulation approach.

## Results

### Consolidated Standards of Reporting Trials

**Figure 2:**
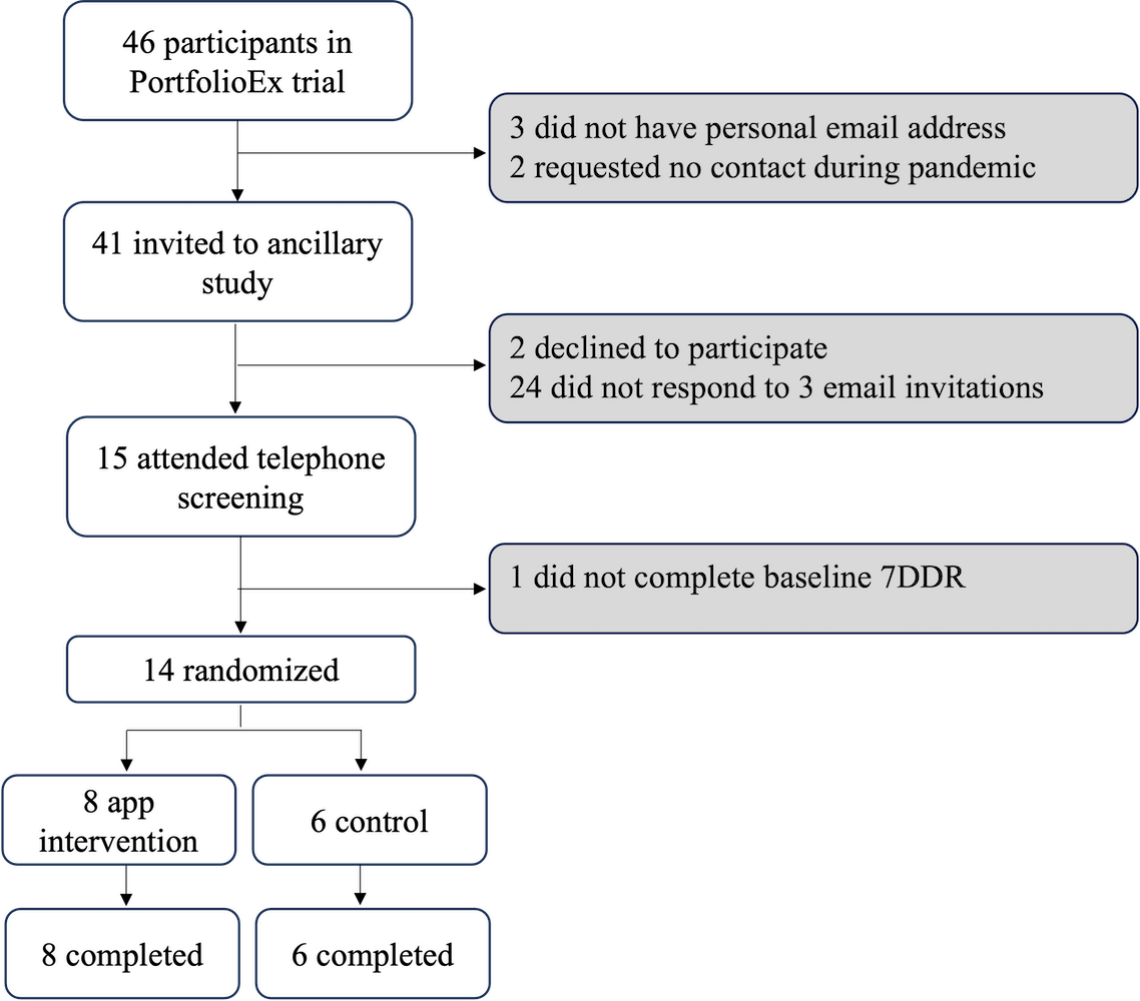
CONSORT (Consolidated Standards of Reporting Trials) flow diagram showing participant flow through the ancillary study. PortfolioEx trial: the Combined Portfolio Diet and Exercise Study; 7DDR: 7-day diet record.

Figure 2 shows the CONSORT diagram of participants in the ancillary study. While a total of 66 participants were randomized to the PortfolioDiet.app group arm in the PortfolioEx trial, 14 dropped out before the ancillary study began. Once REB approval was received, 6 participants had completed the trial or were scheduled to complete the trial within 3 months. Therefore, of the remaining 46 participants, 41 were eligible (3 did not have a personal email and 2 requested no contact during the COVID-19 pandemic). Potential participants were invited by email to participate in the ancillary study. Between July 2021 and February 2022, of the 15 participants who completed a telephone screen, 14 had baseline dietary data and were randomized (intervention group: n=8; control group: n=6) and completed the study. The average duration that the 14 participants had been enrolled in the PortfolioEx trial and were receiving the Portfolio Diet intervention at the Toronto site was 24.6 (SD 4.1; range 18-33) months.

### Baseline Characteristics

Table 1 shows the baseline characteristics of the 14 randomized participants. Participants were primarily female (n=9, 64%), identified mostly as White (n=7, 50%) followed by South Asian (n=3, 21%), Filipino (n=2, 14%), and Black (n=2, 14%). Their mean age was 65 (SD 4, range 52-79) years; 71% (10/14) were on lipid-lowering medication and 29% (4/14) had type 2 diabetes. Adherence to the Portfolio Diet was high in both groups at baseline with a c-PDS of 53% (13.2/25) in the app group and 55% (13.7/25) in the control group. A total of 2 participants (1 in the app group and 1 in the control group) did not provide their final 12-week 7DDR. Therefore, they were excluded from the primary analysis.

**Table 1:** Baseline characteristics of participants.

		Total (N=14)	App group (n=8)	Control group (n=6)	*P* value
Age (y), mean (SD)		65.4 (9)	65 (9)	66 (9)	.96
**Sex**, **n (%)**					.30
	Female	9 (64)	4 (50)	5 (83)	
	Male	5 (36)	4 (50)	1 (17)	
**Race or ethnicity**, **n (%)**					.99
	Black	2 (14)	1 (12.5)	1 (17)	
	Filipino	2 (14)	1 (12.5)	1 (17)	
	South Asian	3 (21)	2 (25)	1 (17)	
	White	7 (50)	4 (50)	3 (50)	
Body weight (kg), mean (SD)		73.1 (13.3)	68.9 (11.3)	78.7 (14.8)	.18
BMI (kg/m^2^), mean (SD)		28.0 (4.3)	26.2 (4.5)	30.3 (5.1)	.07
Waist circumference (cm), mean (SD)		96.6 (11.5)	92.1 (10.7)	102.7 (10.3)	.09
**BP^a^ (mm Hg), mean (SD)**					
	Systolic BP	114.8 (16.9)	113.8 (11.7)	116.1 (23.4)	.80
	Diastolic BP	64.9 (11.4)	61 (7.3)	70 (14.5)	.15
Type 2 diabetes, n (%)		4 (29)	2 (25)	2 (33)	.99
**Lipids (mmol/L), mean (SD)**					
	Total cholesterol	4.8 (1.7)	4.6 (1.9)	5.0 (1.4)	.75
	LDL-C^b,c^	2.8 (1.5)	2.7 (1.9)	2.9 (1.1)	.79
	HDL-C^d^	1.4 (0.4)	1.5 (0.3)	1.4 (0.4)	.70
	Non-HDL-C	3.4 (1.6)	3.3 (2.0)	3.5 (1.2)	.83
	Triglycerides	1.3 (0.5)	1.3 (0.6)	1.3 (0.4)	.99
**Medication use**					
	Lipid-lowering medication, n (%)	10 (71)	7 (88)	3 (50)	.25
	Antihypertensive medication, n (%)	9 (64)	5 (63)	4 (67)	.99
	Duration enrolled in the PortfolioEx trial (months), mean (SD)	24.6 (4.1)	23.3 (4.7)	26.5 (2.3)	.15
	c-PDS^e^ (points; range 0 to 25), mean (SD)	13.4 (4.4)	13.2 (3.7)	13.7 (5.8)	.87

^a^BP: blood pressure.

^b^LDL-C: low-density lipoprotein cholesterol.

^c^LDL-C level was calculated using the Friedewald equation [37].

^d^HDL-C: high-density lipoprotein cholesterol.

^e^c-PDS: clinical Portfolio Diet Score.

### Dietary Adherence to the Portfolio Diet

Table 2 shows the dietary adherence to the Portfolio Diet for the full score (c-PDS), which ranges from 0 to 25 points, and the 5 individual components, which range from 0 to 5 points. The primary outcome of dietary adherence to the Portfolio Diet increased by 1.25 (SD 2.8; 1.25/25, 5%) points in the app group (*P*=.28) and 0.19 (SD 4.4; 0.19/25, 1%) points in the control group (*P*=.93), although neither increase was statistically significant (*P*=.62) from baseline and there was no difference between groups. On the basis of our sample size, the effect size (1.06), and the pooled SD (3.69), the estimated power to detect a statistically significant between-group difference was 7.8% (1-β) with an ⍺=.05, so due to the sample size, we were underpowered to detect a significant difference in dietary adherence between groups.

**Table 2:** Dietary adherence to the Portfolio Diet from weighed 7-day diet records, measured using the clinical Portfolio Diet Score (week 0 to week 12)^a^.

	App group (n=7)					Control group (n=5)				
	Week, mean (SD)		Δ^b^, mean (SD)	*P* value^c^	Week, mean (SD)			Δ, mean (SD)	*P* value^c^	*P* value^d^
	0	12			0		12			
Nuts and seeds, points	3.4 (1.2)	3.6 (1.6)	0.2 (1.8)	.82	2.8 (1.2)		2.7 (1.6)	–0.1 (2.3)	.92	.82
Plant protein, points	2.8 (1.1)	2.6 (1.3)	–0.2 (0.8)	.54	3.2 (1.7)		2.9 (2.3)	–0.3 (0.7)	.48	.91
Viscous fiber, points	3.3 (1.5)	2.8 (1.8)	–0.5 (0.9)	.21	2.6 (1.7)		2.2 (1.2)	–0.4 (0.9)	.32	.94
Plant sterols, points	2.0 (1.8)	3.6 (1.8)	1.6 (1.9)	.08	3.5 (1.7)		4.3 (0.5)	0.7 (1.8)	.41	.46
High MUFA^e^ oils and foods, points	1.6 (1.1)	1.8 (1.2)	0.2 (0.9)	.56	1.6 (1.8)		1.9 (1.6)	0.3 (0.8)	.48	.91
Total c-PDS^f^, points	13.2 (3.7)	14.5 (5.1)	1.3 (2.8)	.28	13.7 (5.8)		13.9 (5.2)	0.2 (4.4)	.93	.62

^a^The individual components are shown in points (range 0 to 5), which make up the total c-PDS (range 0 to 25).

^b^Δ represents change.

^c^*P* value for within group.

^d^*P* value for across groups.

^e^MUFA: monounsaturated fatty acid.

^f^cPDS: clinical Portfolio Diet Score.

### Acceptability

Multimedia Appendix 4 shows the average PortfolioDiet.app use by intervention month. Participants logged into the app an average of 18 (SD 14) days per month over the 12-week intervention period with the number of log-ins trending down over the duration of the intervention but these results were not statistically significant (Table S1 in Multimedia Appendix 5). The average SUS score was 80.9 (SD 17.3), which surpasses the usability quality benchmark threshold of 70, indicating a high level of usability [35]. Table S2 in Multimedia Appendix 5 shows the scores for individual SUS items. The individual responses to the SUS items (range 1-5) show that most participants felt confident using the app (mean 4.0, SD 1.31), they thought the app was easy to use (mean 4.25, SD 1.16), and they felt that the various functions in the PortfolioDiet.app were well linked together (mean 4.5, SD 0.76). Table S3 in Multimedia Appendix 5 summarizes the quantitative responses to the questionnaire**.** More than half of the participants (5/8, 63%) agreed that using the app increased their knowledge about the Portfolio Diet. Tip sheets and email reminders were ranked as the top app features for helping participants learn about the diet and support their interest or engagement in using the app, respectively.

### Participant Insights From Open-Ended Questions

#### Overview

**Figure 3:**
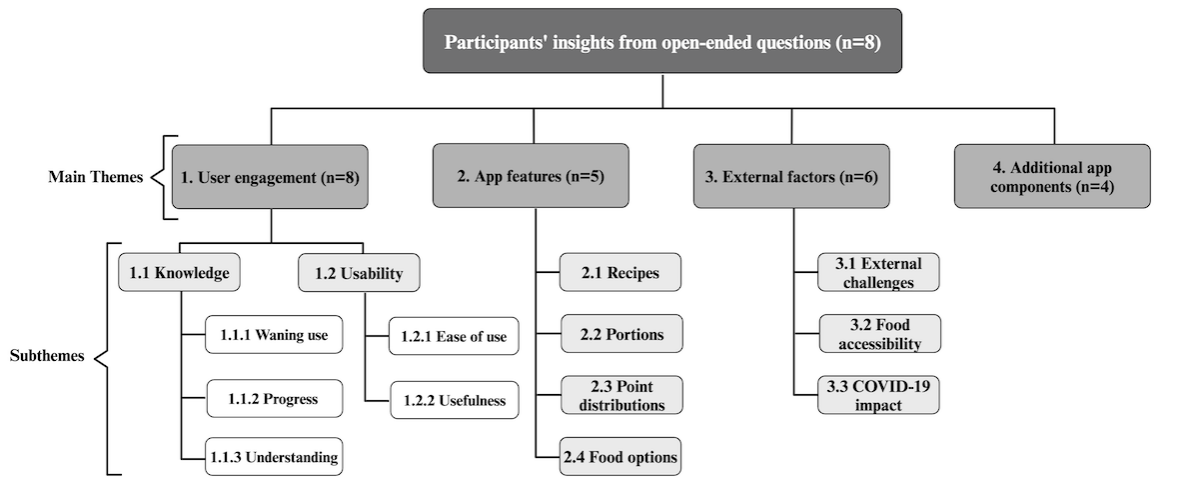
Overview of main themes and subthemes identified from open-ended question responses. The number of participants with statements in each main theme is indicated by “(n=)”.

Figure 3 presents the results of the qualitative data assessments of open-ended questions. The open-ended questions expanded upon the SUS, providing contextual insights into participants’ responses. A total of 4 main themes were identified: user engagement, app features, external factors, and added components. Each theme was further categorized into subthemes. Table S4 in Multimedia Appendix 5 presents individual participant quotations categorized under these themes related to their experience using the PortfolioDiet.app. Notably, 1 participant’s insights were excluded from the table as their questionnaire responses were retrieved through a telephone conversation, wherein a member of the research team documented the responses. However, the insights provided by this participant were considered during data analysis.

#### Theme 1: User Engagement

##### Overview

The theme user engagement describes participants’ experiences using the PortfolioDiet.app and sheds light on how they actively used, responded to, and integrated the app into their lives. Within this overarching theme, we found that participants described their engagement in various ways that could be divided into two subthemes: (1) knowledge, relating to participants’ knowledge acquisition on the Portfolio Diet, which was further subdivided into waning use, progress, and understanding, and (2) usability, relating to the usability of the PortfolioDiet.app, which was further subdivided into usefulness and ease of use.

##### Knowledge

###### Waning Use

It relates to how participants’ engagement with the app transformed over time, revealing a pattern of gradual decline. Some participants mentioned that as they became more acquainted with the Portfolio Diet principles, their initial enthusiasm diminished. This sentiment of diminished engagement appeared to be rooted in the perception that the app’s educational value was more pronounced during the early stages of app use:

I think the app is for new users. After you get up to speed and figure out how to do the [Portfolio Diet] and how [to] split your portions throughout the day, I can’t see using the app daily for me.

Participant 6

###### Progress

Most participants acknowledged the app’s role in helping them learn about their progress on the Portfolio Diet. Some participants referenced the leaderboard feature as being insightful in tracking their progress and understanding where their Portfolio Diet Score (PDS) stands. One participant expressed that the tracking or progress monitoring feature of the PortfolioDiet.app provided them with a sense of being actively engaged in their progress:

I enjoy tracking as it keeps me on target for food intake.

Participant 3

Another participant mentioned that the leaderboard encouraged them to “cheat more rather than eat more [Portfolio Diet] foods.” However, other participants appreciated the app’s tracking and progress monitoring features as they contributed to a sense of accountability and competition, motivating participants to align their dietary choices with the Portfolio Diet principles.

###### Understanding

Participants commented on how the app enriched their comprehension of the Portfolio Diet. Some participants articulated how the app’s clear instructions and visual aids enhanced their understanding of the diet. One participant emphasized the ingenuity of the app’s concept and its thoughtful design:

I think the concept is very clever and built in a meaningful way.... I have a much better understanding of the diet and how I am supposed to follow it.

Participant 5

##### Usability

###### Ease of Use

When exploring the app’s usability, participants elaborated on their impressions of the app’s user friendliness. Participants largely found the app intuitive and easy to use. One participant noted that they had been following the Portfolio Diet for 3 years before incorporating the app into their routine. They found that using the app for tracking purposes was more convenient and practical compared to using a traditional paper checklist:

I was already on the third year of the Portfolio Diet when I started using the app. For me, it was easier [and] more handy to track using the app than using a checklist on paper.

Participant 4

Others mentioned a learning curve associated with using the app, noting the transition from requiring assistance to gaining confidence in using the app:

I was somewhat worried about the complexity of the app but got over it after the first couple of days of trying it out.

Participant 5

Other participants echoed a similar sentiment in their feedback regarding uncertainties about specific aspects of the PortfolioDiet.app. For instance, 1 participant provided positive feedback about the weekly questions for points, but voiced confusion over the meaning of star points and their implications:

The weekly questions for points were an interesting addition that I liked. I could not figure out what the star points meant when I logged out. I couldn’t find an explanation if you miss a certain number of days or a certain threshold of daily points that you would slide backwards in the 30 day points graph.

Participant 6

Over a telephone interview, a participant also highlighted their concern with some technical features of the app, mentioning that the responsiveness of the bars within the app was slower than desired and reporting occasional log-in issues.

###### Usefulness

The usefulness of the PortfolioDiet.app was described by participants when evaluating the app’s usability in their daily routines. A participant shared that the app offered them a unique perspective by focusing on helpful ways to enhance their PDS. By incorporating advice from the PortfolioDiet.app into their routine, they were able to make actionable behavior changes. As described by this participant, adding the liquid plant sterol supplement to their breakfast routine was an easy and impactful way to increase their PDS by 5 points:

[The app] helped me look at how to increase my daily [Portfolio Diet] score. For example, after I started using the app, I got into a regular use of the [plant] sterol supplement with my oatmeal every morning. My use of these supplements was more sporadic but using the app made me appreciate the high value of the supplement.

Participant 5

Alternatively, other participants mentioned they felt that the PortfolioDiet.app did not provide any additional incentives beyond their regular one-on-one meetings with trained dietitians, as part of the ongoing PortfolioEx trial:

There was nothing more in the app than what we were taught to do.

Participant 2

#### Theme 2: App Features

After reviewing the feedback provided by participants, it became evident that several features of the PortfolioDiet.app were prominently mentioned. Specifically, participants emphasized the recipes, portions, point distribution (PDS), and food options.

##### Recipes

Notably, regarding recipes, one participant found them enjoyable to try, while another appreciated the app's inclusion of recipes but did not find that they aligned with their eating style. One participant described the recipes as a “nice addition” but mentioned that they did not try any of them:

The recipes were a nice addition however, I am a simple eater and didn’t try any of the recipes. It is difficult to assess how one of my recipes or a vegan recipe book could be converted so I just assume if it has lots of oat bran or soy within, then it fits with the Portfolio diet.

Participant 6

Conversely, a different participant provided constructive feedback, suggesting that a review of the recipes might be beneficial, as they noted instances where certain ingredients or complete instructions were missing.

##### Portions

The participant feedback encompassed a range of viewpoints regarding the portion sizes recommended by the PortfolioDiet.app. While 1 participant described the portion sizes as “helpful,” others voiced concerns that they appeared “enormous,” “confusing,” and seemingly tailored for a “higher calorie diet”:

Initially, the app portion sizes were confusing .... Some portions on the app (i.e., barley) appeared enormous and put me off.

Participant 6

Two participants drew comparisons between the traditional paper checklist from the PortfolioEx trial they used to track their adherence to the Portfolio Diet and the app’s portion feature, detailing the hurdles they encountered during the learning process. In addition, they emphasized discrepancies between the app’s portion feature and their accustomed checklist. One of the participants described the following:

I did not like that it didn’t line up exactly with the Daily checklist sheets which I used for about a year or more and got used to the portions and amounts on these sheets. It didn’t line up. I also didn’t like at first that I couldn’t change it to my caloric intake.

Participant 7

##### Point Distribution (PDS)

Participants commented on how the point distribution component of the PortfolioDiet.app enabled them to monitor their scores, identify if they were high or low, and explore opportunities to improve their scores through changing aspects of their diet in accordance with the Portfolio Diet principles. One participant described the following:

The app was most helpful in delineating the different categories and how to improve your score if you were low in one of the five categories.

Participant 5

Alternatively, the same participant described how the organization of the point distribution components “frustrated” them as they did not align with the portion sizes they usually ate, evident in the following statement:

However, I found myself to be a little frustrated in some of the way the points are distributed. Using the viscous fibre category as an example that [highlights] the frustration I manage to eat at least an orange or an apple a day but not 2. Also, I eat a fair bit of ... eggplant but never 4 cups worth in one sitting.

Participant 5

Furthermore, another participant shared their experience of confusion while calculating points, expressing uncertainty about the value of food servings in terms of points:

At times, it is confusing calculating points. An example is the Oils. For 1 tsp of oil is the point “1” or “2” points?

Participant 3

##### Food Options

The feedback received consisted mainly of participant approval of the selection of foods included in the PortfolioDiet.app. However, 1 participant articulated desiring a broader range of food choices in the PortfolioDiet.app:

I hope one day the app can be used to track more foods to the categories.

Participant 5

In addition, another participant expressed contentment with the app’s food options, attributed to the convenience of locating these items at the grocery store.

#### Theme 3: External Factors

On the basis of the analysis of the participant’s feedback from the intervention arm, external factors were identified as one of the main themes. External factors explored influences mentioned by participants that either positively or negatively impacted their ability to follow the Portfolio Diet but were not related to the app.

##### External Challenges

Participants mentioned some barriers in following the Portfolio Diet that were not directly related to the app or the COVID-19 pandemic. One participant expressed that the act of traveling posed challenges in adhering to the Portfolio Diet recommendations. While not elaborated upon, this sentiment highlights the real-world implications of dietary interventions, where external factors such as travel can impact the ability to follow dietary interventions:

Travelling makes it more difficult to follow [the Portfolio Diet].

Participant 2

A different participant expressed experiencing fatigue from adhering to the intervention. The participant’s remark indicates that maintaining adherence to the Portfolio Diet can become challenging over time. This insight underscores the potential external factors, such as lack of novelty, that can influence an individual’s engagement with this dietary intervention:

It’s me getting tired of following a vegan diet.

Participant 4

##### Food Accessibility

Comments on the practicality of accessing recommended foods for the Portfolio Diet were captured as an important area for understanding how the Portfolio Diet can be applied to diverse populations. A participant shared that they use soy foods and shelf-stable soy milk from a particular store, likely due to the convenience it offered. They also mentioned finding an alternative plant sterol powder at a specific store, which they incorporated into their diet. This account provides valuable insight into the participant’s resourcefulness in adapting their dietary habits to the Portfolio Diet, especially when faced with challenges like limited availability of certain products:

I find soy foods in the freezer aisle of Loblaws and use the shelf life Soy milk so I don’t have to go to the store so often during Covid... I found a [plant] sterol powder at Healthy Planet that substitutes for the [plant] sterol margarine that’s no longer produced and it’s good in shakes or in my all-bran buds cereal ....

Participant 6

While the only comment made in this study about food accessibility was positive, we emphasize future work on the Portfolio Diet to capture future participants’ feedback on this subtheme.

##### COVID-19 Pandemic Impact

As this study was run during the COVID-19 pandemic, a specific open-ended question related to its impact on the participants was included within the questionnaire. Understanding how participants from various situations experienced the COVID-19 pandemic and how it impacted their adherence to the Portfolio Diet may influence interpretation of the results of the study. Participants mentioned issues related to a lack of in-person meetings with the study dietitians and gym closures, while others articulated how they had been self-sufficient and were able to find study foods independently outside of the clinic. Interestingly, as the study was at the “tail end” of the lockdown, the impact of business reopening was noted by 1 participant:

Yes, with lock down, I was able to follow the diet very well, but since opening up, I have been more inclined to eat out and also crave foods that I haven’t had in a long time at my favorite restaurants.... Definitely have felt some slow down in my incentive to keep strictly to the diet since the reopening. Also we are travelling a bit and I am excited to try the foods of the region we are travelling in so I also strayed from the Portfolio regime as a result.

Participant 7

#### Theme 4: Added App Components

Participants articulated suggestions for app improvements and several requests, including the ability to record half portions, more food suggestions, visual meal plans, and more information related to diabetes. A participant pointed out the app’s lack of capability for personalized adjustments to their dietary plan, which the dietitians had been able to offer them individually. This feedback underscores the value of personalized guidance and highlights a potential area for improvement in the app’s functionality to better accommodate individualized dietary adjustments:

The app doesn’t allow for personal [tweaking] to the portfolio as the dietitians have been able to do for me personally.

Participant 7

Some recommendations for features were already embedded within the app. As an example, 1 user suggested including the option to record half portions of food, a feature already available on the PortfolioDiet.app. This feedback indicates that the participant was not aware of this feature, suggesting it was not intuitive. Overall, we found that there were no overlapping suggestions from participants, demonstrating the importance of ensuring the app can be personalized to any user based on their needs and preferences.

## Discussion

### Principal Findings

We conducted a 12-week randomized controlled ancillary mixed methods study to assess the effect of the PortfolioDiet.app on dietary adherence and its acceptability among high-risk adults. Although adherence was higher for the PortfolioDiet.app group after 12 weeks (ie, increased by 1.25/25, 5% and 0.19/25, 1% in the app group and control group, respectively), no difference between the groups was observed in this small ancillary study.

The PortfolioDiet.app was rated as usable, with the app surpassing the usability quality benchmark threshold [35]. While participants engaged often with the app over the 12 weeks, use gradually declined. Beyond the usability, the app increased self-reported knowledge of the Portfolio Diet. The demonstration of increased knowledge in those who had already been learning about the Portfolio Diet for an average of approximately 2 years further supports the acceptability of the app in this high-risk population. These results shed light on the potential of app-based technology as a promising platform to translate the Portfolio Diet to adults at high CVD risk.

The decline in use combined with the trending increase in adherence to the Portfolio Diet from 7DDRs, aligns with the intended purpose of the app as an educational tool aimed at fostering users’ self-efficacy. As participants become more knowledgeable and confident in applying the principles of the Portfolio Diet, it is expected that their reliance on the app and use of the tracking progress feature would gradually decrease. However, based on participant feedback, modifications to the app to make this expectation clear to the user may further improve app acceptability. This messaging could include a note on the role the app can play for users at various times in their life, when they perhaps fall off the diet and need support to return to following the Portfolio Diet.

The qualitative data assessments complemented the quantitative findings. Analysis of open-ended questions identified 4 primary themes that encapsulated participants interactions with the PortfolioDiet.app. Among the themes, “user engagement” underscores the dynamic interactions participants had with the app, their knowledge gained, and the integration of its features into their routine. This was also evident in the quantitative findings which revealed that most participants felt that various functions of the PortfolioDiet.app were well linked together. The app’s usefulness for self-monitoring of dietary adherence was noted as important and helpful by some participants. The educational aspect of the app was a recurrent point of mention among participants, with several of them noting how it enhanced or aided their current understanding of the Portfolio Diet. This observation aligns with the quantitative finding where more than half of the participants said that the app increased their knowledge of the Portfolio Diet. On the other hand, comments suggesting that the app provided no new information beyond what was provided in their regular one-on-one meetings with trained dietitians may provide an indication of why others may have responded “No” to this question about increasing knowledge on the Portfolio Diet. As all participants had already been participating in the PortfolioEx trial learning about the Portfolio Diet, this finding suggests the app is reinforcing counseling from dietitians.

The second theme, “app features,” highlighted features participants found helpful or frustrating. These findings align with the current understanding as self-tracking and gamification features have been found as successful tools in health apps for behavior change [41]. However, some features of the app, such as the portions, could be better explained by using pop-up windows with additional instructions or through other modifications to the app.

The theme “external factors” delved into influences beyond the app’s control on dietary adherence. Notably, the impact of the COVID-19 pandemic was explored, revealing its implications on participants’ adherence patterns as pandemic restrictions shifted.

The fourth theme “additional app components” covered participants’ feedback to include additional features to the app. Participants expressed a desire for additional food options and visual meal plans, as well as more diabetes-related information. Other desirable app modifications can be distilled from comments relating to the dislike of certain features (eg, leaderboard), challenges in logging foods, and adding half portion sizes. These comments imply possible modifications to the app that could improve its usability and acceptability, such as features of the app that need to be more intuitive and the ability for users to customize their own targets and dashboard.

Identifying that tip sheets and videos supported learning and engagement in the app can be leveraged in addressing some of the challenges identified by participants. Tip sheets could be developed to include tips while traveling or on the go, for meal plan ideas, and further support for those with diabetes. Integrating an interactive frame within the app to showcase new content, such as tip sheets, as well as videos to further support engagement may be a useful modification based on the participant feedback. Taken together, these findings suggest that the PortfolioDiet.app has the potential to support participants in adhering to the Portfolio Diet and is considered acceptable by adults at high CVD risk.

### Comparison With Prior Work

This study is the first to use the PortfolioDiet.app in high-risk adults. While health apps have seen widespread adoption, findings have been inconsistent when looking at their effects on behavior change and health outcomes. Similar to our findings, a systematic review and meta-analysis of 47 studies revealed that web-based interventions targeting risk factors show promise in reducing CVD risk, yet their effects were moderate and waned over time [42]. Inconsistencies in effects may be related to differences in the app features, the participant’s health status, and whether the app intervention has been tailored to the population.

Apps that target dietary behavior change have also shown promise with suggestion that in those with chronic disease, use of health apps with nutrition components improved health outcomes, with 64% of studies showing sustained behavior change for 6 to 12 months [43]. These conclusions differ from others who found health benefits were only observed in short-term studies (less than 6 months), suggesting that secondary prevention participants may be more motivated to make sustained behavior change.

When looking at health apps focused on delivering a therapeutic dietary pattern, a systematic review of 5 studies in participants with hypertension or prehypertension, found that mobile apps providing the Dietary Approaches to Stop Hypertension diet were associated with higher adherence to this diet and lower blood pressure when compared to controls [44]. However, the authors could not pinpoint the most effective features of these apps from a users’ perspective. Identifying specific features may not be entirely possible as different population groups may prefer different strategies [43], emphasizing the importance of tailoring health apps to their intended population and allowing for personalization within the app. Interestingly, qualitative analysis of other health apps have identified similar themes with “new features” being identified as 1 of the 3 themes in adolescences with knee pain [45], mirroring our theme “Added app components.” Without specific prompts, this shared interest underscores a patient’s desire to shape tools meant to assist them and the importance of involving them in the cocreation process.

Several qualitative studies have identified barriers to nutrition app use. König et al [46] found that app usability was important for sustained uptake. The PortfolioDiet.app has been deemed usable in both a convenience sample of users and in our current representative sample of participants. When comparing our usability score to others in the literature, a raw SUS score of 80 would be better than 75% of all apps tested; however, average SUS scores varies based on the type of app being tested [47]. A systematic review of health apps found an average SUS score of 76.6 (SD 15.12), but when excluding physical activity apps, the average SUS dropped to 68.1 (SD 14.05) [48]. This finding aligns with the general understanding that nutrition apps are challenged with usability issues [46]. Specific to nutrition, an analysis of the top 7 diet-tracking apps (from iOS iTunes and Android Play web-based stores) found an average SUS of 70.9 (SD 12.72) with a range from 46.7 to 89.2, after 3 undergraduate nutrition students used the apps over a 2-week period [49].

In addition, personalized and tailored educational material, reminders, progress tracking, and goal setting have been found to be highly valued features [50], all of which are present in the PortfolioDiet.app. The usability and knowledge acquisition demonstrated in this study also aligns with the results of a previous quality assessment study of the PortfolioDiet.app in a convenience sample of users [26].

### Strengths and Limitations

The primary strength of this study is the assessment of the PortfolioDiet.app within its intended target population of adults at high risk of CVD, allowing for modifications to the app to support its use in the intended users. The collection of both quantitative and qualitative data is also a strength of this study as it allowed for a comprehensive understanding of participants’ experiences with the PortfolioDiet.app. In addition, the synergy between the SUS findings along with the insights derived from qualitative analysis, where participants largely found the app intuitive and easy to use, strengthens our confidence that the app was considered usable by this study population. The influence of the COVID-19 pandemic on participants’ experiences and engagement underscores the significance of remote health care solutions in ensuring quality care delivery despite challenging circumstances.

A major limitation was the restricted pool of participants, exacerbated by delays in the REB review due to the COVID-19 pandemic, among other challenges experienced by the research community [51]. These challenges led to a sample below the estimated necessary sample size, with the estimated power to detect a statistically significant between-group difference being 7.8% (1–β), ⍺=.05, so we were underpowered to detect a significant difference in dietary adherence between groups. The limited sample size should also be considered when interpreting the qualitative findings. While data saturation may be achievable with relatively small samples (9-17 interviews) [39], our sample falls below this range, so a cautious interpretation of the results is necessary.

In addition, we did not measure health-related risk factors directly. While much of the research in the realm of health apps has shown improvements in behaviors, there remains a notable gap in the literature concerning their impact on intermediate risk factors and other health outcomes. Consequently, it is imperative that future research endeavors incorporate assessments of health outcomes, such as lipid profiles, to provide a more comprehensive understanding of the impact of these apps on health and disease outcomes.

In addition, in light of research findings suggesting that marginalized populations may also experience digital exclusion exacerbating existing health disparities, it is crucial to emphasize the necessity of future research involving underserved groups [52].

Finally, the use of the SUS is another limitation as it was not originally tailored for evaluating health apps. However, the 100-point scale facilitates clear communication to nonexperts in the field. Moreover, the concise nature of the SUS, featuring 10 questions, ensures swift participant completion and reduces response burden, which is especially important when participants are not visiting the study center and instead are completing the questionnaires remotely. Possibly related to its high ease of use, the SUS was used in 40 of the 96 studies in a scoping review of health apps in older (>65 years) individuals [53]. Although other questionnaires to assess the usability of mobile health (mHealth) apps have recently been developed, the SUS remains widely used and considered suitable for assessing digital health apps [48,54]. However, to enhance specificity to mHealth apps, future evaluations of the PortfolioDiet.app administering questionnaires could include the user-oriented Mobile Application Rating Scale or the recently validated mHealth App Usability Questionnaire, which includes additional questions to integrate feedback on app features [55,56].

### Implications and Future Directions

As CVD continues to be a leading cause of mortality in Canada and globally [57], prioritizing lifestyle interventions for disease prevention and management is pivotal. Among these interventions, the Portfolio Diet is an effective therapy for managing dyslipidemia and reducing the risk of CVD. As a tool for disseminating this nutrition therapy, the PortfolioDiet.app may serve to increase the adoption of the Portfolio Diet.

Notably, there is growing interest among older adults in using mobile apps to support their learning efforts. In a survey conducted among Canadian retired older adults (aged >55 years), 78.5% agreed or strongly agreed that mobile devices made their learning easier [58], highlighting the potential of the PortfolioDiet.app to engage and empower older individuals, who are a critical demographic for cardiovascular health management. This observation underscores the substantial implications of the PortfolioDiet.app and the importance of tailoring the app to ensure older adults can engage with the app. From this study, we can discern both the app’s strengths and limitations in its intended population of high-risk adults. These insights will guide us in refining the PortfolioDiet.app, creating a tool that better meets the needs of its target population. Subsequent work will incorporate the feedback received through modification to the design of the PortfolioDiet.app. While this work was undertaken in older high-risk adults, further research is needed in more diverse and underserved populations.

### Conclusions

This small ancillary study suggests the PortfolioDiet.app is considered acceptable, easy to use, and increases knowledge of the Portfolio Diet in adults at high CVD risk. The present findings highlight the potential of the PortfolioDiet.app as an educational tool, reinforcing counseling from dietitians. In general, participants appreciated the app’s self-monitoring features as they contributed to a sense of accountability, motivating participants to align their dietary choices with the Portfolio Diet principles. Future refinements to ensure the app is intuitive and its features are well explained and can be personalized could enhance participant engagement and adherence to the Portfolio Diet for improved cardiovascular health. We await the results of a randomized controlled trial investigating the effect of the PortfolioDiet.app on lipid targets in a high-risk population, which may provide evidence of its potential health benefits.

## Appendix

Multimedia Appendix 1 CONSORT 2010 checklist.

Multimedia Appendix 2 CONSORT-eHEALTH checklist (V 1.6.1).

Multimedia Appendix 3 PortfolioDiet.app participant feedback questionnaire.

Multimedia Appendix 4 Average days logged into the PortfolioDiet.app over the intervention (12 weeks; n=8).

Multimedia Appendix 5 Supplemental tables including use, usability, and feedback on the PortfolioDiet.app.

Multimedia Appendix 6 Full list of all disclosures.

## Abbreviations

7DDR: 7-day diet record
CONSORT: Consolidated Standards of Reporting Trials
c-PDS: clinical Portfolio Diet Score
CVD: cardiovascular disease
LDL-C: low-density lipoprotein cholesterol
mHealth: mobile health
PDS: Portfolio Diet Score
REB: research ethics board
SUS: System Usability Scale

## Data Availability

The datasets generated or analyzed during this study are available from the corresponding author on reasonable request.
