# Supplementary material for: Acceptability of a Web-Based Health App (PortfolioDiet.app) to Translate a Nutrition Therapy for Cardiovascular Disease in High-Risk Adults: Mixed Methods Randomized Ancillary Pilot Study
Source: JMIR Cardio. 2025 Mar 28;9:e58124. doi: 10.2196/58124 (PMC11992491; doi:10.2196/58124)
Supplement: Multimedia Appendix 3 [file cardio_v9i1e58124_app3.docx]

# Portfolio Diet App Participant Feedback Questionnaire

--- Beginning of Questionnaire ---
 **Please answer the following questions below and then send it back to us by email. We hope to get your honest feedback about your experience with using the Portfolio Diet App. Your feedback will be used to improve the Portfolio Diet App for future research.**

**The 10 following questions are asking about how easy you found using the app was. Please add an “X” in the brackets.** Please do not think about the question for a long time, just provide your first response to each question. If you feel you cannot respond to a particular question, please mark the centre box of the scale.

1. I think that I would like to use this app frequently (often)

Strongly disagree [    ] [    ] [    ] [    ] [    ] Strongly agree

2. I found the app unnecessarily complex

Strongly disagree [    ] [    ] [    ] [    ] [    ] Strongly agree

3. I thought the app was easy to use

Strongly disagree [    ] [    ] [    ] [    ] [    ] Strongly agree

4. I think that I would need the support of a technical person to be able to use this app

Strongly disagree [    ] [    ] [    ] [    ] [    ] Strongly agree

5. I found the various functions in this app were well integrated (linked together)

Strongly disagree [    ] [    ] [    ] [    ] [    ] Strongly agree

6. I thought there was too much inconsistency (mismatch) in this app

Strongly disagree [    ] [    ] [    ] [    ] [    ] Strongly agree

7. I would imagine that most people would learn to use this app very quickly

Strongly disagree [    ] [    ] [    ] [    ] [    ] Strongly agree

8. I found the app very cumbersome (hard) to use

Strongly disagree [    ] [    ] [    ] [    ] [    ] Strongly agree

9. I felt very confident using the app

Strongly disagree [    ] [    ] [    ] [    ] [    ] Strongly agree

10. I needed to learn a lot of things before I could get going with this app

Strongly disagree [    ] [    ] [    ] [    ] [    ] Strongly agree

**The following 8 questions are more specific to this dietary study.** If you are uncomfortable answering any of the questions, please feel welcome to skip them by writing *NA*.

1. Did you increase your knowledge about the Portfolio Diet while using the Portfolio Diet app?  Please add an “X” in the brackets.

[    ] Yes

[    ] No

[    ] Not sure

2. Please expand on your answer above, and write your answer below each question.

           If yes, what did you learn?

            If no, why do you think the app did not increase your knowledge?


3. Was there anything you particularly enjoyed or did not like about the app? Please write your answer below.


4. Which app characteristic helped you learn about the diet the most? Please rank them as 1 (for the best) to 4 (as the worst).  Please add your numbers in the brackets.

[    ] recipes

[    ] tip sheets

[    ] videos 
[    ] infographic

5. Which app characteristic supported your interest/ engagement in using the app the most? Please rank them as 1 (for the best) to 7 (as the worst).  Please add your numbers in the brackets.   

[    ] star rewards

[    ] recipes

[    ] tip sheets

[    ] videos
[    ] email reminders
[    ] 30-day points graph

[    ] leaderboard

6. In your home, who purchases the food most often?  Please add an “X” in the brackets. Choose all that apply.

[    ] I do 
[    ] my parent(s)/ caretaker

[    ] my children

[    ] my roommate 
[    ] my spouse or partner

[    ] other, please specify: [          ]

7. In your home, who makes the meals most often?  Please add an “X” in the brackets. Choose all that apply.

[    ] I do 
[    ] my parent(s)/ caretaker

[    ] my children

[    ] my roommate 
[    ] my spouse or partner

[    ] other, please specify: [          ]

8. Has anything (i.e. COVID-19) impacted your ability to follow the Portfolio diet? If yes, please describe by writing your answer below.


--- End of questionnaire ---
