# Supplementary figures and images for: Acceptability of a Web-Based Health App (PortfolioDiet.app) to Translate a Nutrition Therapy for Cardiovascular Disease in High-Risk Adults: Mixed Methods Randomized Ancillary Pilot Study
Source: JMIR Cardio. 2025 Mar 28;9:e58124. doi: 10.2196/58124 (PMC11992491; doi:10.2196/58124)

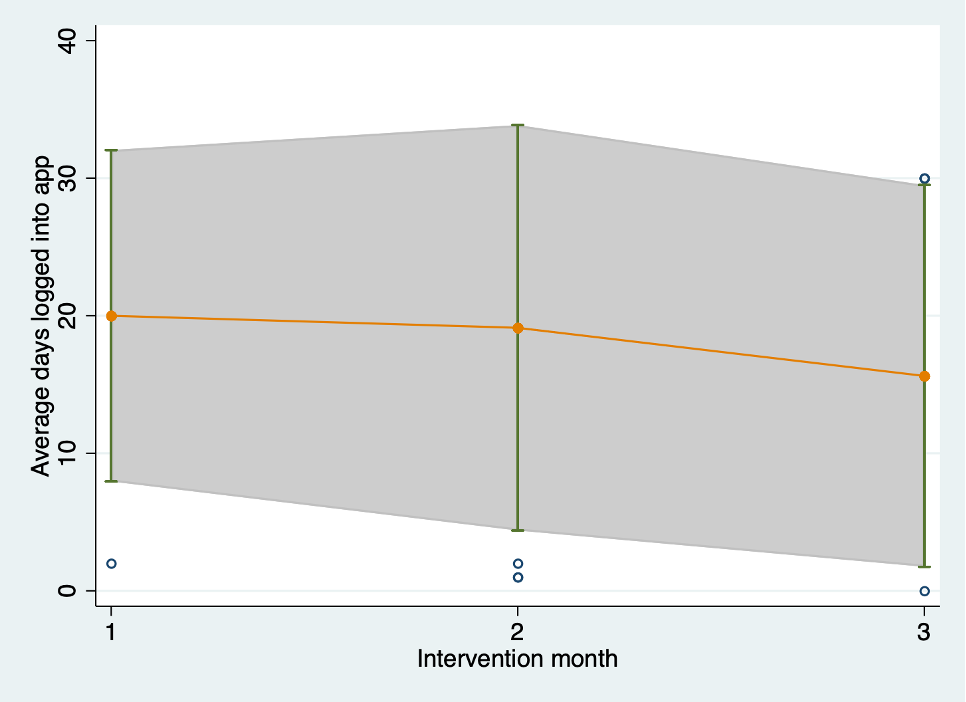

Supplement: Multimedia Appendix 4 [file cardio_v9i1e58124_app4.png]
