# Supplementary material for: Acceptability of a Web-Based Health App (PortfolioDiet.app) to Translate a Nutrition Therapy for Cardiovascular Disease in High-Risk Adults: Mixed Methods Randomized Ancillary Pilot Study
Source: JMIR Cardio. 2025 Mar 28;9:e58124. doi: 10.2196/58124 (PMC11992491; doi:10.2196/58124)
Supplement: Multimedia Appendix 5 [file cardio_v9i1e58124_app5.docx]

# **Supplement Table S1:** Average PortfolioDiet.app usage over 12-week intervention and c-PDS autocalculated by app (week 0 to week 12) shown by week intervals (n=8)

| **Weeks** | **0-4 (month 1)** | **4-8 (month 2)** | **8-12 (month 3)** | **0-12 (average over 3 months)** |
| --- | --- | --- | --- | --- |
| **Days logged** | 19.63 (11.66) | 19.00 (14.92) | 15.63 (13.88) | **18.08 (13.49)** |
| **c-PDS^a^** | 17.16 (3.55) | 17.54 (4.46) | 19.16 (5.06) | **17.86 (4.40)** |
| **Nuts & Seeds^a^** | 3.22 (1.06) | 3.20 (1.27) | 3.83 (1.17) | **3.42 (1.16)** |
| **Plant Protein^a^** | 3.18 (0.79) | 3.22 (1.21) | 3.65 (1.36) | **3.35 (1.12)** |
| **Viscous fibre^a^** | 3.11 (1.13) | 3.07 (1.38) | 3.21 (1.35) | **3.13 (1.29)** |
| **Plant Sterols^a^** | 4.18 (0.72) | 4.28 (1.02) | 4.11 (1.05) | **4.19 (0.93)** |
| **Oils (MUFA) ^a^** | 3.48 (1.01) | 3.77 (1.09) | 4.36 (0.85) | **3.87 (0.98)** |

**Data presented as mean (±SD)**

**^a^**Values shown do not take into account days where nothing was logged.

# **Supplement Table S2:** Scores for individual SUS items from participants randomized to the PortfolioDiet.app intervention (n=8)

| **Question** | **Mean (SD)** |
| --- | --- |
| 1. I think that I would like to use this app frequently (often) | 4.0 (1.31) |
| 2. I found the app unnecessarily complex | 2.38 (1.51) |
| 3. I thought the app was easy to use | 4.25 (1.16) |
| 4. I think that I would need the support of a technical person to be able to use this app | 1.63 (1.19) |
| 5. I found the various functions in this app were well integrated (linked together) | 4.5 (0.76) |
| 6. I thought there was too much inconsistency (mismatch) in this app | 1.5 (0.93) |
| 7. I would imagine that most people would learn to use this app very quickly | 3.88 (1.36) |
| 8. I found the app very cumbersome (hard) to use | 1.88 (1.36) |
| 9. I felt very confident using the app | 4.63 (1.06) |
| 10. I needed to learn a lot of things before I could get going with this app | 1.50 (1.07) |
| **Overall SUS score** | 80.94 (17.37) |

SUS, System Usability Scale

| **Supplement Table S3:** Quantitative responses from feedback questionnaire (week 12) (n=8) | |
| --- | --- |
| **Question** | **Response** |
| Did you increase your knowledge about the Portfolio Diet while using the Portfolio Diet app? (%; yes:no:unsure) | 63:25:12 |
| Which app characteristic helped you learn about the diet the most? (N users) | tip sheets=3, recipes=2, videos=1, Infographic=1 n/a=1 |
| Which app characteristic supported your interest/ engagement in using the app the most? (N users) | email reminders=3 videos=1, tip sheets=1, Star rewards=1, 30-day points graph=1, n/a=1 |
| In your home, who makes the meals most often? (N users) | I do=5, my spouse or partner=3 |
|  |  |

# **Supplement Table S4:** Participant Quotations Categorized Under Themes Related to their Experience using the PortfolioDiet.App

| **Themes** | **Sub themes** | | **Definition** | **Quotes** |
| --- | --- | --- | --- | --- |
| 1. User engagement (n=8) | 1.1 Knowledge | 1.1.1 Waning use | Participant's usage of the app gradually decreased after they initially started using it, and they eventually lost track of using it altogether. Indicating a diminishing level of interest or engagement with the app as time passed. | “Unfortunately, my use of it waned after I learned about my status. So I would say that it was educational but because it was not demanded I lost track of using it.”  ***(participant 5)***  “I think the app is for new users. After you get up to speed and figure out how to do the [Portfolio Diet] and how [to] split your portions throughout the day, I can’t see using the app daily for me. Using it for a specific period […] would be doable but not daily for an extended period of time. After 30 days of using the app and figuring out how it worked, I found that I just wanted to mark my foods by using the bar graph rather than enter my foods eaten into the app. I was happy to be finished using the app at the 3-month mark.”  ***(participant 6)*** |
|  |  | 1.1.2 Progress | How the app aids in monitoring participants’ adherence and progress | “I think the app put a quantitive value to my participation factor. In other words, I could see that I was at least an “average” follower of the diet. I think it was helpful to see how other participants were and see that I was on the spectrum. In other words, there were other dieters who were below and above me in their total score. The app was most helpful in delineating the different categories and how to improve your score if you were low in one of the five categories. I like the app because I think it helped me to gain a better understanding of what the diet is about and what I need to eat more of. I would say that after using it for a week I learned about where I stood in the spectrum of other participants. More specifically my scores hovered around 16-18 occasionally maybe reaching a maximum of 20 points on one or two of the days.” ***(participant 5)***  “The app was most helpful in delineating the different categories and how to improve your score if you were low in one of the five categories.” ***(participant 5)***  “I enjoy tracking as it keeps me on target for food intake.” ***(participant 3)***  “Nothing has impacted my ability to follow the Portfolio diet. The app is a great asset to keep me focused on making healthy choices.” ***(participant 3)***  “I think the leaderboard encouraged me to cheat more - rather than eat more [Portfolio Diet] food” ***(participant 2)*** |
|  |  | 1.1.3 Understanding | App’s role in enhancing participants’ comprehension of the Portfolio Diet | “I like the app because I think it helped me to gain a better understanding of what the diet is about and what I need to eat more of.” ***(participant 5)***  “I think your instructions and simple diagrams did a good job of educating the user.” ***(participant 5)***  “I think the concept is very clever and built in a meaningful way. […] I have a much better understanding of the diet and how I am supposed to follow it.” ***(participant 5)***  “Learned that it has some pictures and clear things to help me learn about the diet.” ***(participant 1)***  “The learning modules that explained each category of the diet and portion size was helpful.” ***(participant 6)***  “There was nothing more in the app than what we were taught to do.” ***(participant 2)***  “I read the booklets and handouts that were given to me at the beginning, and find most of my knowledge comes from the nutritionists/dietitians who support me.”  ***(participant 7)*** |
|  | 1.2 Usability | 1.2.1 Usefulness | Comments about the overall usefulness of the app | “What was not clear was how the information was being used or processed. If it was for my education then it did it’s job in the first week and then it was just not all that useful for me. My diet is pretty much consistent for breakfast and lunch. So in a sense it helped me look at how to increase my daily score. For example, after I started using the app, I got into a regular use of the sterol supplement with my oatmeal every morning. My use of these supplements was more sporadic but using the app made me appreciate the high value of the supplement.” ***(participant 5)***  “There was nothing more in the app than what we were taught to do” ***(participant 2)***  “I think the leaderboard encouraged me to cheat more - rather than eat more PD food” ***(participant 2)***  “The app doesn't allow for personal tweaking to the portfolio as the dietitians have been able to do for me personally.” ***(participant 7)***  “I also didn't like at first that I couldn't change it to my caloric intake.” ***(participant 7)*** |
|  |  | 1.2.2 Ease of Use | Participants’ impressions of the app’s user-friendliness | “It was easy to use.” ***(participant 5)***  “I was somewhat worried about the complexity of the App but got over it after the first couple of days of trying it out.” ***(participant 5)***  “[…] the developers were quick to respond to questions. I like the fact that it is all built in.” ***(participant 5)***  “The first time you need someone’s help. Once you know its not very difficult to get going except when changing. I started eating okra and chickpeas, it was hard to change in the app.” ***(participant 1)***  “Super easy and I love the favourite meal feature to precode my breakfast” ***(participant 2)***  “[I enjoy the] ease of use, [it is] portable, [has] bright colours, [is] easy to read and use, [I] like the points system” ***(participant 7)***  “[…] it is hard to access the app. I don't have a convenient way to access it still. Definitely could use some one-on-one training on the app.”  ***(participant 7)***  “I really liked the colour codes and the ease of use. Sometimes I didn't like the two steps to get into the app itself.” ***(participant 7)***  “I was already on the third year of the Portfolio diet when I started using the app. For me, it was easier/more handy to track using the app than using a checklist on paper.” ***(participant 4)***  ***“***The weekly questions for points were an interesting addition that I liked. I could not figure out what the star points meant when I logged out. I couldn’t find an explanation if you miss a certain number of days or a certain threshold of daily points that you would slide backwards in the 30 day points graph. I sort of figured that out myself. Maybe that was a video that I didn’t watch.” ***(participant 6)*** |
| 2. App features (n=5) | 2.1 Recipes | | Participants’ feedback about the included recipes in the app | “The recipes need to be reviewed as some of the ingredients or full instructions are missing.” ***(participant 3)***  “Trying the recipes was very enjoyable.” ***(participant 3)***  “The recipes were a nice addition however, I am a simple eater and didn’t try any of the recipes. It is difficult to assess how one of my recipes or a vegan recipe book could be converted so I just assume if it has lots of oat bran or soy within, then it fits with the Portfolio diet.”  ***(participant 6)*** |
|  | 2.2 Portions | | Comments related to portion sizes and measurements on the app | “Initially, the app portion sizes were confusing as I was already using the original Portfolio diet checklist portions. Converting from the Portfolio checklist portions to the app portions was not easy as I had already learned the original way. Some portions on the app (i.e., barley) appeared enormous and put me off. Having the ability to record half portions or incremental portions should be considered. ***(participant 6)***  “The learning modules that explained each category of the diet and portion size was helpful.” ***(participant 6)***  “[…] the required amounts of foods per pillar are based on a higher calorie diet. The portions do not directly correspond to the checklists that I had been using for over 1.5 years and it was confusing to relearn and remeasure.” ***(participant 7)***  “I did not like that it didn't line up exactly with the Daily checklist sheets which I used for about a year or more and got used to the portions and amounts on these sheets. It didn't line up. I also didn't like at first that I couldn't change it to my caloric intake.” ***(participant 7)*** |
|  | 2.3 Point distribution (c-PDS) | | Participants’ thoughts on the allocation of points in the app | “The app was most helpful in delineating the different categories and how to improve your score if you were low in one of the five categories. […] I would say that after using it for a week I learned about where I stood in the spectrum of other participants. More specifically my scores hovered around 16-18 occasionally maybe reaching a maximum of 20 points on one or two of the days.”  ***(participant 5)***  “My diet is pretty much consistent for breakfast and lunch. So in a sense it helped me look at how to increase my daily score. For example, after I started using the app, I got into a regular use of the sterol supplement with my oatmeal every morning. My use of these supplements was more sporadic but using the app made me appreciate the high value of the supplement.” ***(participant 5)***  “However, I found myself to be a little frustrated in some of the way the points are distributed. Using the viscous fibre category as an example that [highlights] the frustration I manage to eat at least an orange or an apple a day but not 2. Also I eat a fair bit of eat eggplant but never 4 cups worth in one sitting. I may have a bit of a dish that I made that lasts me all week and yet I have no way of recording the amount that I consume daily. I am sure there will be ways that you will find to address this issue. Same goes for oranges. I have one orange a day pretty just about every day. But I couldn’t find a way to log in my one orange. Such issues can be discouraging if you are thinking the app should be used on a regular basis.” ***(participant 5)***  “I think the app put a quantitive value to my participation factor. In other words, I could see that I was at least an “average” follower of the diet. I think it was helpful to see how other participants were and see that I was on the spectrum. In other words, there were other dieters who were below and above me in their total score.” ***(participant 5)***  “At times, it is confusing calculating points. An example is the Oils. For 1 tsp of oil is the point “1” or “2” points?” ***(participant 3)***  “The weekly questions for points were an interesting addition that I liked. I could not figure out what the star points meant when I logged out. I couldn’t find an explanation if you miss a certain number of days or a certain threshold of daily points that you would slide backwards in the 30 day points graph. I sort of figured that out myself. Maybe that was a video that I didn’t watch. I think the app is for new users. After you get up to speed and figure out how to do the diet and how you split your portions throughout the day, I can’t see using the app ‘daily’ for me.”  ***(participant 6)***  “[I] like the points system” ***(participant 7)*** |
|  | 2.4 Food options | | Comments related to the variety of foods in the app’s categories | “I hope one day the app can be used to track more foods to the categories.” ***(participant 5)***  “I find soy foods in the freezer aisle of Loblaws and use the shelf life Soy milk so I don’t have to go to the store so often during Covid… I found a sterol powder at Healthy Planet that substitutes for the sterol margarine that’s no longer produced and it’s good in shakes or in my all-bran buds cereal (mixed with a little cinnamon to prevent white floaties).” ***(participant 6)*** |
| 3. External factors (n=6) | 3.1 External challenges | | Barriers faced by participants in following the Portfolio Diet not directly related to the app | “Travelling makes it more difficult to follow” ***(participant 2)***  “It's me getting tired of following a vegan diet.”  ***(participant 4)*** |
|  | 3.2 Food accessibility | | Comments on the practicality of accessing recommended foods | “I find soy foods in the freezer aisle of Loblaws and use the shelf life Soy milk so I don’t have to go to the store so often during Covid… I found a sterol powder at Healthy Planet that substitutes for the sterol margarine that’s no longer produced and it’s good in shakes or in my all-bran buds cereal […]” ***(participant 6)*** |
|  | 3.3 COVID-19 impact | | Comments related to COVID-19 and app usability | “During Covid, I was very self-sufficient and did not need to go into the clinic to obtain more of the diet foods. The sterol liquid packets are really convenient vs the powder that I use (it mixes in milk better) but I did not stop by the clinic to get more. To battle any mental health depression, my jogging program makes me feel great for about 5 hours after each of my 4 runs per week.” ***(participant 6)***  “Yes, with lock down, I was able to follow the diet very well, but since opening up, I have been more inclined to eat out and also crave foods that I haven't had in a long time at my favorite restaurants […]. Definitely have felt some slow down in my incentive to keep strictly to the diet since the re-opening. Also we are travelling a bit and I am excited to try the foods of the region we are travelling in so I also strayed from the Portfolio regime as a result.” ***(participant 7)***  “No, maybe exercise has been a little challenging as the gym has been closed.” ***(participant 1)*** |
| 4. Additional app components (n=4) | Feedback on potential new features or improvements to the app | | | “Why not consider adding the ‘exercise’ component to the app and that way all the information is contained in one space” ***(participant 5)***  “I hope one day the app can be used to track more foods to the categories.” ***(participant 5)***  “Having the ability to record half portions or incremental portions should be considered” ***(participant 6)***  “the app doesn't allow for personal [tweaking] to the portfolio as the dietitians have been able to do for me personally” ***(participant 7)*** |
