# Supplementary material for: Acceptability of a Web-Based Health App (PortfolioDiet.app) to Translate a Nutrition Therapy for Cardiovascular Disease in High-Risk Adults: Mixed Methods Randomized Ancillary Pilot Study
Source: JMIR Cardio. 2025 Mar 28;9:e58124. doi: 10.2196/58124 (PMC11992491; doi:10.2196/58124)
Supplement: Multimedia Appendix 6 [file cardio_v9i1e58124_app6.docx]

### **Multimedia Appendix 6:** Complete list of disclosures

**MEK** was a part-time employee at INQUIS Clinical Research, Ltd., a contract research organization.

**LC** has received research support from the Canadian Institutes of Health Research (CIHR), Protein Industries Canada (a government of Canada Global Innovation Clusters), Alberta Pulse Growers, and the United Soybean Board (USDA soy Checkoff program).

**AJG** has received travel support and/or honoraria from Vinasoy, the Soy Nutrition Institute Global, and the Academy of Nutrition and Dietetics.

**SA-C** has received an honorarium from the International Food Information Council for a talk on artificial sweeteners, the gut microbiome, and the risk for diabetes.

**AZ** is a part-time research associate at INQUIS Clinical Research, Ltd, a contract research organization. She has received consulting fees from the Glycemic Index Foundation.

**CWCK** has received grants or research support from the Advanced Food Materials Network, Agriculture and Agri-Foods Canada (AAFC), Almond Board of California, Barilla, Canadian Institutes of Health Research (CIHR), Canola Council of Canada, International Nut and Dried Fruit Council, International Tree Nut Council Research and Education Foundation, Loblaw Brands Ltd, the Peanut Institute, Pulse Canada and Unilever. He has received in-kind research support from the Almond Board of California, Barilla, California Walnut Commission, Kellogg Canada, Loblaw Companies, Nutrartis, Quaker (PepsiCo), the Peanut Institute, Primo, Unico, Unilever, WhiteWave Foods/Danone. He has received travel support and/or honoraria from the Barilla, California Walnut Commission, Canola Council of Canada, General Mills, International Nut and Dried Fruit Council, International Pasta Organization, Lantmannen, Loblaw Brands Ltd, Nutrition Foundation of Italy, Oldways Preservation Trust, Paramount Farms, the Peanut Institute, Pulse Canada, Sun-Maid, Tate & Lyle, Unilever and White Wave Foods/Danone. He has served on the scientific advisory board for the International Tree Nut Council, International Pasta Organization, McCormick Science Institute and Oldways Preservation Trust. He is a founding member of the International Carbohydrate Quality Consortium (ICQC), Chair of the Diabetes and Nutrition Study Group (DNSG) of the European Association for the Study of Diabetes (EASD), is on the Clinical Practice Guidelines Expert Committee for Nutrition Therapy of the EASD and is a Director of Glycemia Consulting and the Toronto 3D Knowledge Synthesis and Clinical Trials foundation.

**DJAJ** has received research grants from Loblaw Companies Ltd., the Almond Board of California, Soy Nutrition Institute (SNI), and the Canadian Institutes of Health Research (CIHR). He has received in-kind supplies for trials as a research support from the Almond board of California, Walnut Council of California, American Peanut Council, Barilla, Unilever, Unico, Primo, Loblaw Companies, Quaker (Pepsico), Pristine Gourmet, Bunge Limited, Kellogg Canada, WhiteWave Foods. He has been on the speaker's panel, served on the scientific advisory board and/or received travel support and/or honoraria from Lawson Centre Nutrition Digital Series, 19th Annual Stare-Hegsted Lecture, 2024 Diabetes Canada Conference, Nutritional Fundamentals for Health (NFH)-Nutramedica, Saint Barnabas Medical Center, The University of Chicago, 2020 China Glycemic Index (GI) International Conference, Atlantic Pain Conference, Academy of Life Long Learning, the Almond Board of California, Canadian Agriculture Policy Institute, the Loblaw Companies Ltd, the Griffin Hospital (for the development of the NuVal scoring system), the Coca-Cola Company, Epicure, Danone, Diet Quality Photo Navigation (DQPN), Better Therapeutics (FareWell), Verywell, True Health Initiative (THI), Heali AI Corp, Institute of Food Technologists (IFT), Soy Nutrition Institute (SNI), Herbalife Nutrition Institute (HNI), Herbalife International, Pacific Health Laboratories, Nutritional Fundamentals for Health (NFH), the Soy Foods Association of North America, the Nutrition Foundation of Italy (NFI), the Toronto Knowledge Translation Group (St. Michael's Hospital), the Canadian College of Naturopathic Medicine, The Hospital for Sick Children, the Canadian Nutrition Society (CNS), and the American Society of Nutrition (ASN). He is a member of the International Carbohydrate Quality Consortium (ICQC). His wife, Alexandra L Jenkins, is senior scientist of INQUIS Clinical Research Inc. (Clinical Research Organization), his 2 daughters, Wendy Jenkins and Amy Jenkins, have published a vegetarian book that promotes the use of the low glycemic index plant foods advocated here, The Portfolio Diet for Cardiovascular Risk Reduction (Academic Press/Elsevier 2020 ISBN:978-0-12-810510-8)and his sister, Caroline Brydson, received funding through a grant from the St. Michael's Hospital Foundation to develop a cookbook for one of his studies.

**JLS** has received research support from the Canadian Foundation for Innovation, Ontario Research Fund, Province of Ontario Ministry of Research and Innovation and Science, Canadian Institutes of health Research (CIHR), Diabetes Canada, American Society for Nutrition (ASN), National Honey Board (U.S. Department of Agriculture [USDA] honey “Checkoff” program), Institute for the Advancement of Food and Nutrition Sciences (IAFNS), Pulse Canada, Quaker Oats Center of Excellence, INC International Nut and Dried Fruit Council Foundation, The United Soybean Board (USDA soy “Checkoff” program), Protein Industries Canada (a Government of Canada Global Innovation Cluster), Almond Board of California, European Fruit Juice Association, The Tate and Lyle Nutritional Research Fund at the University of Toronto, The Glycemic Control and Cardiovascular Disease in Type 2 Diabetes Fund at the University of Toronto (a fund established by the Alberta Pulse Growers), The Plant Protein Fund at the University of Toronto (a fund which has received contributions from IFF among other donors), The Plant Milk Fund at the University of Toronto (a fund established by the Karuna Foundation through Vegan Grants), and The Nutrition Trialists Network Fund at the University of Toronto (a fund established by donations from the Calorie Control Council, Physicians Committee for Responsible Medicine, and Login5 Foundation). He has received food donations to support randomized controlled trials from the Almond Board of California, California Walnut Commission, Danone, Nutrartis, Soylent, and Dairy Farmers of Canada. He has received travel support, speaker fees and/or honoraria from FoodMinds LLC, Nestlé, Abbott, General Mills, Nutrition Communications, International Food Information Council (IFIC), Arab Beverage Association, International Sweeteners Association, Calorie Control Council, Phynova, and Collaborative CME and Research Network (CCRN). He has or has had ad hoc consulting arrangements with Almond Board of California, Perkins Coie LLP, Tate & Lyle, Ingredion, and Brightseed. He is on the Clinical Practice Guidelines Expert Committees of Diabetes Canada, European Association for the study of Diabetes (EASD), Canadian Cardiovascular Society (CCS), and Obesity Canada/Canadian Association of Bariatric Physicians and Surgeons. He serves as an unpaid member of the Board of Trustees of IAFNS. He is a Director at Large of the Canadian Nutrition Society (CNS), founding member of the International Carbohydrate Quality Consortium (ICQC), Executive Board Member of the Diabetes and Nutrition Study Group (DNSG) of the EASD, and Director of the Toronto 3D Knowledge Synthesis and Clinical Trials foundation. His spouse is a former employee of Nestle Health Science and AB InBev.

**SMQ, GV, KR, NA, MP, SSP, DP, SMG, RGJ,** and **VSM** report no relevant competing interests.
